# Reduced quality and accelerated follicle loss with female reproductive aging - does decline in theca dehydroepiandrosterone (DHEA) underlie the problem?

**DOI:** 10.1186/1423-0127-20-93

**Published:** 2013-12-13

**Authors:** Judith H Ford

**Affiliations:** 1 Centre for Rural Health and Community Development, University of South Australia, Adelaide 5000, South Australia

**Keywords:** Ageing, Aging, DHEA, Fat metabolism, Trisomy, Aneuploidy, Follicular reserve, Theca, PPARα, Ceramide

## Abstract

Infertility, spontaneous abortion and conception of trisomic offspring increase exponentially with age in mammals but in women there is an apparent acceleration in the rate from about age 37. The problems mostly commonly occur when the ovarian pool of follicles is depleted to a critical level with age but are also found in low follicular reserve of other etiologies. Since recent clinical studies have indicated that dehydroepiandrosterone (DHEA) supplementation may reverse the problem of oocyte quality, this review of the literature was undertaken in an attempt to find an explanation of why this is effective?

In affected ovaries, oxygenation of follicular fluid is low, ultrastructural disturbances especially of mitochondria, occur in granulosa cells and oocytes, and considerable disturbances of meiosis occur. There is, however, no evidence to date that primordial follicles are compromised. In females with normal fertility, pre-antral ovarian theca cells respond to stimulation by inhibin B to provide androgen-based support for the developing follicle. With depletion of follicle numbers, inhibin B is reduced with consequent reduction in theca DHEA. Theca cells are the sole ovarian site of synthesis of DHEA, which is both a precursor of androstenedione and an essential ligand for peroxisome proliferator-activated receptor alpha (PPARα), the key promoter of genes affecting fatty acid metabolism and fat transport and genes critical to mitochondrial function. As well as inducing a plethora of deleterious changes in follicular cytoplasmic structure and function, the omega 9 palmitate/oleate ratio is increased by lowered activity of PPARα. This provides conditions for increased ceramide synthesis and follicular loss through ceramide-induced apoptosis is accelerated.

In humans critical theca DHEA synthesis occurs at about 70 days prior to ovulation thus effective supplementation needs to be undertaken about four months prior to intended conception; timing which is also suggested by successful interventions to date. In humans and primates that undergo adrenarche, the adrenal zona reticularis (ZR) is the major site of DHEA production, however this is also reduced with age. Concomitant loss in function of the ZR might account for the acceleration in the rate of aging seen in humans in the late thirties’ age group.

## Review

Maternal aging is associated with a dramatic increase in infertility, a high risk of miscarriage and of giving birth to live-born infants with Down’s syndrome. As women increasingly defer child bearing, this biological problem is becoming a growing social and health economic issue. Reproductive aging has been extensively studied during the last fifty years, initially most intensively by human geneticists and more recently by scientists and clinicians working with assisted conception. Nevertheless despite the huge input no one explanation has fitted all the data.

Part of the problem in trying to explain a biological phenomenon of this type is that the evidence crosses many sub-disciplines. Today, as research techniques become more refined and the evidence generated more complex, silos of knowledge are generated and experts find it difficult to cross between sub-disciplines. This paper will attempt to coordinate the information from several areas to find a logical model from the data that is currently available.

### The problem of reproductive aging

**Figure 1:**
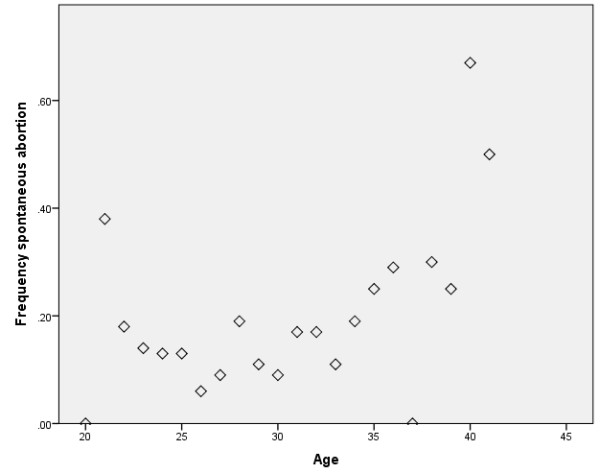
**Graph of the frequency of spontaneous abortion by age, amongst pregnancies monitored in a group of normal women who had no interventions and were studied prospectively from the time they attempted to conceive in a study known as the PALS: Pregnancy and Lifestyle Study.**

Initially, extensive international data showed fairly consistent age-related risks of Down’s syndrome live-births in different ethnic groups [1]: the rate was about 1/1490 at age 20–24, 1/200 at 35, 1/60 at 40 and 1/11 at 49. The observed trisomic births, however, were soon found to be only the *tip of the iceberg*. Older women have an extremely high rate of pregnancy loss of both chromosomally normal and abnormal conceptions and increased rates of spontaneous abortions occur at similar ages as the Down syndrome births (Figure 1); moreover spontaneously aborted conceptions that are chromosomally abnormal are mostly trisomic [2]. Recent large studies using assisted reproductive technology have confirmed that aneuploidy is a leading impediment to reproduction in older women [3].

Many cytogenetic-based hypotheses have been put forward to explain the mechanisms underlying the generation of maternal age trisomies and many of these are summarised [4,5]. Various aspects of meiosis appear to be adversely affected by the aging process but no one mechanism explains all the data. The impression is that there is a multiply determined deterioration of the meiotic process with aging. Some have proposed that this is caused by an age-dependent system of gamete selection where the last remaining follicles are inherently defective but there is no mechanism outlined for such selection.

Although the disturbances of meiosis and the consequent chromosomal abnormalities have attracted the most attention of geneticists seeking to explain the underlying mechanisms, all cytogenetic studies of age-related pregnancy losses also showed high rates of loss of chromosomally normal conceptions. These chromosomally normal embryos appeared to show the same range of developmental abnormalities and developmental arrest as those that were chromosomally abnormal [6,7], which suggests that the underlying problem of the aging oocyte may be cytoplasmic rather than nuclear in origin. There is no doubt that genetic imbalances are responsible for many specific defects however many of the developmental abnormalities found in both chromosomally normal and abnormal abortuses could be caused by disruptions to cell division (both meiotic and mitotic), secondary to abnormalities of structure and function of cytoplasmic organelles, and the consequences on cell number, size and distribution.

### Ultrastructure, cytology and gene expression of granulosa cells and oocytes in normal and aging ovaries prior to ovulation

The competence of granulosa cell mitochondria is critical to follicle survival and function. Detailed studies of the ultrastructure of resting follicles in younger, aged 27–32, and older, aged 38–45 women showed statistically significant age-related changes in the cytoplasmic organelles of the granulosa cells, including changes in the density of the mitochondrial matrix, the frequency of dilated smooth endoplasmic reticulum and Golgi complex [8]. When granulosa cells from peri-ovulatory follicles of younger (aged 20–32) and older (aged 38–41) women attending an IVF program were compared, the expression of mRNA and protein levels from the mitochondrial genes superoxide dismutases Cu and Zn *SOD1*, Mn *SOD* (*SOD2*) and catalase were all significantly decreased in the older women [9]. Ultrastructural studies of the granulocytes from the older women showed structurally defective mitochondria and fewer lipid droplets in 63.3% of cells. By comparison, only 8.2% of cells of the younger women showed ultrastructural defects.

In resting follicles (oocyte size about 900 μm), oocytes of older women show a significant increase in the fraction of vacuoles compared with younger women and whilst the nuclear and cytoplasmic membranes and number of microvilli showed no apparent difference between younger and older women, the proportion of mitochondria showing a high density matrix was highly significantly increased with age [8].

The possibility of mitochondrial dysfunction playing a key role in a cytoplasmic-based cause of age-related problems is further supported by recent studies in aging mice oocytes that demonstrate that mitochondria are both morphologically abnormal and are decreased in abundance [10]. Furthermore, studies of oxidative phosphorylation in individual bovine oocytes showed that aged oocytes have reduced maximum respiratory capacity [11] and aging meiosis II oocytes in mice and hamsters have lower levels of ATP [12]. These studies all support earlier work that found that the adenosine triphosphate (ATP) content of human oocytes is correlated with developmental potential and outcome after in vitro fertilization [13].

Some researchers have considered that cumulus cell function may be a more major determinant than the oocyte itself of the quality of the mitochondria in the activated oocytes. In elegant studies undertaken to determine which cumulus genes were both induced by the LH peak and critical to oocyte competence, mRNA expression in cumulus cells was prospectively studied in human oocytes that would be used for intracytoplasmic sperm injection [14]. Gene functions that were up-regulated in cumulus cells were identified in mature oocytes (meiosis II compared to Germinal vesicle (GV) stage cells) and those that were up-regulated in cumulus cells from mature oocytes that yielded a blastocyst at day 5/6 of *in vitro* culture. In all, 23 up-regulated functions were identified, one of which was ‘fatty acid biosynthesis’ and in a previous paper where they studied the expression of six key genes, the same group specifically identified that both *delta*-*9 desaturase* and *delta*-*5 desaturase* were up-regulated in top quality embryos at day 2 [15]. Nevertheless, these same genes had lower mean expression in the fertilized oocytes that produced high quality embryos after 5–6 days of culture than in those that had low grade embryos, suggesting that in the optimum follicles, the regulation of these and other genes are under tight control. In a somewhat similar study, proteomics was used to study 1423 cumulus proteins in the follicles of younger and older women [16]. These authors found age-related differences in 7.7% of the levels of protein expression and the majority of the genes that differed between age-groups were involved with metabolism, oxidative phosphorylation and post-transcriptional mechanisms.

The results of all the ultrastructural and biochemical studies make it clear that the structure and function of the cytoplasmic organelles, especially the mitochondria, of both the oocytes and the granulosa cells, which are critical to fertilization and normal embryonic development, are markedly compromised in the oocytes of older women prior to ovulation. The studies do not, however, prove that the primordial follicles, held in the dictyotene stage of meiosis, are in any way compromised nor that there is any complex system of follicle selection as has been proposed in some of the previous hypotheses [4,5]. Since studies have not yet been undertaken it is possible that the primordial follicles are already compromised however, if they are not compromised, then there is an age-determined and follicle reserve size effect on oocyte development that is responsible for the disturbed function and deleterious outcomes that affects follicles during development from primordial follicle to about 900 μm size.

### DHEA supplementation for low follicular reserve

Possible therapeutic effects from DHEA supplementation were first suggested by Casson [17]. Since then many IVF clinics have tested regimes in which they supplement older women and/or those with diminished ovarian reserves with DHEA. Extensive clinical work has been undertaken by Gleicher and Barrad [18] and many of the published results have recently been reviewed [19]. The latter authors concluded that while several studies show improvements in pregnancy rates, large randomized prospective trials are still needed. Current studies differ in the dosages of DHEA given and seem to be rather haphazard in the duration of treatment given. To date treatments that are most successful use interventions of about 12 weeks and this suggests that deficiency of DHEA might exert its affects on the earliest stages of follicle growth, possibly at the pre-antral stage.

One of the greatest difficulties in assessing the current treatments is the mix of patients used. For example, one recent study used only ‘poor responders’ [20] and achieved no increase in clinical pregnancies. Despite this, the study showed that after three months of daily treatment with 75 mg DHEA, participants had increased retrieval of mature oocytes and dramatic reduction in follicular fluid hypoxic inducible factor 1 (HIF-1) levels. The authors suggested that the best reproductive outcomes from DHEA treatment in poor responders may be due to the effect on the follicular microenvironment. By comparison a ‘self-controlled’ study of 32 women with various causes of infertility including age, using the same DHEA treatment, had both increased numbers of retrieved oocytes and increased fertilization rates [21]. It thus seems that there are positive effects from treatment with DHEA but that the treatment might not be appropriate for all patients as apart from aging, many other different mechanisms can cause reduction of the follicular reserve.

### Ovarian theca, androgens and reproductive aging

#### Androgens and key roles in ovarian steroidogenesis and effects of age

Theca cells have been referred to as the forgotten cell of the ovarian follicle [22] and yet they play a pivotal role in providing both structural and androgen-based hormonal support to the developing follicles: they are the sole ovarian site for the production of dehydroepiandrosterone (DHEA) and androgens. To date there has been a dearth of studies published on the earliest theca function and aging in normal women possibly because studies have focussed on the biochemical and cellular events closer to the time of ovulation. It is for this reason that the following study is described in detail.

Female macaques are similar to women in having a 28 day menstrual cycle, a natural menopause following a period of decline in follicle numbers and a similar hormonal profile. Thus Ethun [23] used the cynomolgus macaque as a model to study theca function and the relationship between androgen synthesis and aging The relative expression of the androgenic enzymes P450c17 and Cytb5 versus 3βHSD were measured by immunofluorescence and used to characterise androgen synthesis in the different cell layers at each stage in follicle growth in each of 26 monkeys. High expression of the 3βHSD enzyme competes with P450c17 such that if 3βHSD is high, pregnenolone is converted into progesterone but in the absence of 3βHSD, P450c17 is converted into DHEA. In each monkey, one ovary was used to determine the total number of primordial follicles while the other was used for immunohistological studies. The major finding with respect to aging was that theca DHEA and other androgen production was influenced by follicle number: immunostaining for androgenic enzymes in the theca interna layer of growing follicles was reduced in all ovaries with lower numbers of follicles. Androgenic enzymes were only expressed in the theca cells, not the granulosa cells, and the androgenic enzymes P450c17 and CYTB5 were first expressed in the theca cells at the advanced secondary follicle stage (≥ 150 μm) and then more intensely in the antral follicles.

In the advanced secondary follicles only P450c17 and CYTB5 were expressed. In antral follicles, the staining of each of these enzymes was increased in intensity but 3βHSD was also expressed at a low level. Thus DHEA is first produced in theca cells of advanced secondary follicles. Studies using the same enzyme localisation technique in human follicles gave similar evidence of early synthesis of DHEA by theca cells but staining was not observed in follicles smaller than 200 μm [24,25]. Neither of these groups performed age-related studies.

Theca DHEA has a direct role in androgen synthesis and is converted in the theca cells to androstenedione which is in turn transported to the granulosa cells where after binding to the androgen receptor, it is converted into estrone and 17β estradiol [22]. Just as importantly, however, theca DHEA stimulates the production of peroxisome proliferator-activated receptor alpha (PPARα) and in rat ovaries PPARα expression is limited to the theca and stromal cells [26]. Moreover, DHEA is a ligand for PPARα and is necessary for gene induction by DHEA [27]. In general, PPARα plays a key role in fatty acid metabolism and is highly expressed in mammalian tissues such as the heart, brown adipose tissue, kidney, intestine and the liver [28] and white adipose tissue [29]. PPARα promotes fatty acid transport across membranes, including into mitochondria, as well as up-regulating delta-9 (also known as stearoyl CoA- desaturase 1) and delta-6 desaturases (the key enzymes involved in fatty acid desaturation and elongation in the Omega 3,6 and 9 pathways) and genes critical to mitochondrial function [30]. Indeed in humans and mice, PPARα regulates some 240 target genes, which include delta-6 and delta-9 desaturases and the majority, if not all, genes involved in mitochondrial oxidative phosphorylation [31]. Reduced expression of PPARα in the aging theca is probably sufficient to account for the initiation of the subsequent cytoplasmic dysfunction observed in aging oocytes.

The effect of DHEA treatment on mitochondrial function has been demonstrated in both rat liver and brain where it significantly stimulated many mitochondrial enzymes and up-regulated oxidative energy metabolism [32], presumably through activation of PPARα. Furthermore, PPARα is upregulated in response to oxidative stress [33] and it is likely that PPARα upregulation accounts for the reduction in HIF-1 levels in DHEA treated ‘poor responders’ [20].

#### Low androgens: ceramide production and acceleration of follicle loss through apoptosis

Ceramide, a major component of the lipid bilayer of cell membranes, can participate in cellular signalling. In aging oocytes in Drosophila, disturbances of ceramide and its transport protein (CERT) were found to contribute to disturbances of structure and function of mitochondria [34]. In mice, ceramide was found in all the cumulus cells in old mice whereas it was rare, present in only about 5% of the cumulus cells of young mice. When present, ceramide is translocated from the cumulus cells to the oocytes through gap junctions and in older mice, spikes in ceramide levels, lead to apoptosis [35]. Using primary mouse myoblast cultures, [36] have shown that dihydroceramide desaturase (DESI), the enzyme that controls the synthesis of ceramide, is dependent on palmitate. Higher levels of cellular palmitic acid increase mRNA encoding DESI whereas co-treatment with oleate prevents the increase in ceramide.

Delta-9 desaturase, one of the key enzymes regulated by PPARα, is the key enzyme involved in the conversion of palmitic acid to oleic acid. When levels of this enzyme are low, the levels of palmitic acid will rise relative to oleic acid, conditions that promote the production of ceramide and hence conditions that promote apoptosis and accelerated loss of follicles.

#### Age and pre-antral development

The time taken by human follicles to develop from the pre-antral follicle of about 150 μm to the mature follicle of 1500–2000 diameter μm is about 70 days [37]. If the earliest stages of theca development and DHEA expression are important to the normal development of the follicle, and these commence at about the 200 μm size, then by the time oocytes reach 900 μm (the size used in the ultrastructural studies above), they have already been exposed to an androgen-dependent cellular environment for a considerable period of time. Indeed since age-related or follicle-depleted altered levels of androgens might be expected to have major effects on the development and function of the cytoplasmic organelles in the two to three months prior to ovulation, any successful treatments of age-related theca dysfunction would need to occur within this period.

### Age-related rates –mathematical models

One outstanding feature of the trisomy data is its age-related rates, which show a relatively gradual rate of increase up to about age 37 and then greatly accelerate. The complexity of the rates has been discussed by many authors but was first studied in some detail by Penrose e.g. [38] and then taken up at length by Hook e.g. [39]. Authors argued that there was either a statistical artefact or that there was likely to be more than one parameter determining the rate, perhaps a maternal age independent and a maternal age dependent component?

It was predicted [40] that oocyte aging could be estimated from the size of the ovarian follicular store, once that could be measured. Overall, the decline in the size of the pool shows a remarkably consistent relationship with female age and the point of age-related change is essentially identical to those of age-related changes for each of live Down syndrome births and the rates of trisomy. With mathematical modelling Faddy [41] describes the curve of logged total numbers of follicles against age that corresponds to a ‘broken stick’ bi-exponential regression. In this model, there is an obvious acceleration of the rate of follicle loss that starts at about age 38 whereas others propose [42] that the apparent biphasic phenomenon can be better explained by a ‘power model’ that proposes that decay of non growing follicles (NGF) is constantly accelerating. When tested against the ‘power’ model predictions, the following regression values were observed: age r = − 0.80, antral follicle count (AFC) r = 0.78, anti-Mullerian hormone (AMH) r = 0.72, follicle stimulating hormone (FSH) r = − 0.32 and inhibin B (r = 0.40).

The ovarian pool has also been studied in mice [43,44]. In rodents there is also an exponential decrease in the size of the ovarian pool with aging but unlike in humans, there is no evidence of a ‘broken stick’ phenomenon; the rate of aging appears to occur at a consistent rate. Moreover the rates of follicle loss appear to differ between strains.

### Hormonal markers of reproductive aging

It is clear that AMH is the best indicator of follicle numbers but whilst the AMH-specific receptor type II (AMHRII) may be expressed in the theca cells of preantral and antral follicles [45], the major role of AMH seems to be as an inhibitor of recruitment of primordial follicles [46]. AMH is positively correlated with follicle number so levels decline with increasing age and decreasing follicle numbers. Follicle stimulating hormone (FSH) has long been recognised as a marker of reproductive decline and although an imperfect predictor of ovarian response [47] is a reasonable marker of the relationship between reproductive decline and age. FSH levels are positively correlated with age. The correlation with follicle numbers in the model above was only – 0.32 and FSH levels may be more related to inhibin B which plays a critical role in mediating the age-related rise in FSH in older women; neither activin A nor inhibin A seems to show age-related changes [48].

The relationships of Inhibin B and A, FSH, estradiol and progesterone levels to aging were further elucidated in a study that measured the four hormones across the menstrual cycles in younger (less than 35) and older women (35 and older), and in longitudinal studies at 10 year intervals in three women [49]. In these studies both inhibin B and A were observed to decline with age but the decline in inhibin B precedes that of inhibin A and more importantly, the decline in inhibin B precedes the increase in FSH. The reduction in inhibin B was most dramatic when measured in the early and mid-luteal phases.

Androstenedione, the immediate androgen product of DHEA also shows an age-related profile, which over the reproductive years is quite similar to the decline in reproductive competence [50]. Reference values for about twenty participants in each age group showed mean ± SD serum androstenedione values (nmol/l) of - ages 21–25: 5.8 ± 1.7; ages 26–30: 5.2 ± 1.4; ages 31–35: 5.3 ± 2.6; ages 36–40: 4.7 ± 2.2; ages 41–45 (pre-menopausal): 3.4 ± 1.2 and post-menopausal: 3.7 ± 1.3.

### Androgen paracrine regulation by inhibin B and ovarian follicle decline

Inhibin B has been shown to stimulate the synthesis of androgens and proliferation in primary cultures of ovarian theca cells whilst activin inhibits them [51]. Inhibin B is secreted from the granulosa cells of developing preantral follicles and small antral follicles in intact follicles in culture but is not directly stimulated by FSH [52]. The role of inhibin B has been reviewed extensively and it is concluded that inhibin B has paracrine or paracrine-like action, positively upregulating androgen production by ovarian theca cells (e.g. [53]. Furthermore, mutations in the inhibin alpha gene *INHα* are significantly associated with premature ovarian failure [54].

Inhibin B suppresses serum follicle stimulating hormone (FSH) concentration [55] so high levels of FSH always indicate low inhibin B. Although low inhibin B is not the best predictor of follicle number, its decline has been described as a ‘primary event in the aging of the reproductive axis’ [56] and is one of if not the ‘earliest marker of decline in follicle number’ [49]. Moreover, since inhibin B is needed to upregulate DHEA production in theca cells and DHEA plays a key and direct role in theca androgen synthesis then inhibin B almost certainly plays a major role in the regulation of age-related change.

### Relating biochemical changes to the maths

(a) The mammalian system.

The decline with age of inhibin B and the subsequent effects on DHEA, androgen synthesis, PPARα activation and altered regulation of hundreds of genes is sufficient to explain both the effects of aging on decline in quality and pregnancy outcome and the accelerated loss of follicle number that is observed in mammals and is most well defined in rodent models. It does not, however, explain the accelerated decline seen in humans in the late-thirties age group. This requires an extra factor that is absent in rodents and other mammals and indeed in many primates.

(b) Humans and close primate relatives- an additive role of the adrenal cortex zona reticularis?

In humans and those primates that undergo adrenarche, the adrenal cortex zona reticularis (ZR) is the key site of synthesis of DHEA and its sulphate DHEAS. In humans, adrenal synthesis is high during fetal development then falls rapidly after birth. At about age five to six, the levels start to rise again (the stage known as adrenarche) until levels reach a peak at about age 20 [57]. From age 20 onwards in men and women, the synthesis of both dehydroepiandrosterone (DHEA) and its sulphate ester DHEAS decline, although levels in males are almost double those in females [58]. In aging of the adrenal cortex, the decline in the biosynthesis of DHEA and DHEAS are the most striking changes and the simplest, but not yet proven hypothesis, is that it results from a decline in the number of functional zona reticularis (ZR) cells, which secrete it [59]. The age-dependent reduction in the physical size of the ZR and a disruption of its architecture are obvious “although the normal mode by which the size of the adrenal cortex is controlled is only partly understood, it is known to involve feedback via the hypothalamus and pituitary” [59].

Low serum inhibin B, associated with low follicle number may also be a determinant of reduced adrenal ZR function since inhibin B and activin are expressed in the adrenal cortex ZR and play major signalling roles in the ZR [60]. Indeed the alpha sub-unit of inhibin is only expressed in the ZR cells of the adrenal cortex [61]. Whether ACTH is independently involved or whether the age-related physical loss of adrenal ZR cells is the critical factor remains to be determined. However this additional site of loss of production of DHEA could easily account for the sudden acceleration of the age-related deterioration after about age 37. That the ‘broken stick’ graph has thus far only been reported in humans and not in other mammalian models that lack adrenal synthesis of DHEA lends strong weight to this model.

One important limitation to this model is the current lack of information in humans concerning the relative age-related rates of synthesis of DHEA by the ovary and the adrenal glands and whether there is any specific requirement for synthesis within the theca cells. Serum steroid levels from normal females and one female with bilateral adrenalectomy indicated that the ovaries synthesise about 20% of DHEA and that this is maximal at mid-cycle [62]. Similar estimates of the ovarian contribution to DHEA were achieved when DHEA levels were measured before and after dexamethasone suppression of adrenal function [63]. Although the postmenopausal ovary is well known to actively secrete DHEA and androgens [64], to date there is no study that shows the relative contributions that occur from the two organs at different ages in normal women.

The question of whether DHEAS could be used effectively as a pre-hormone for ovarian steroidogenesis was studied using radioactively labelled DHEAS and T. The results demonstrated that plasma DHEAS served as a pre-hormone for 48% of follicular fluid testosterone however, the fractions of androstenedione, estrone and estradiol that were labelled were minimal, each only about 4% [65]. These data suggest that there may indeed be a specific requirement for synthesis of DHEA within the theca cells.

## Conclusions

**Figure 2:**
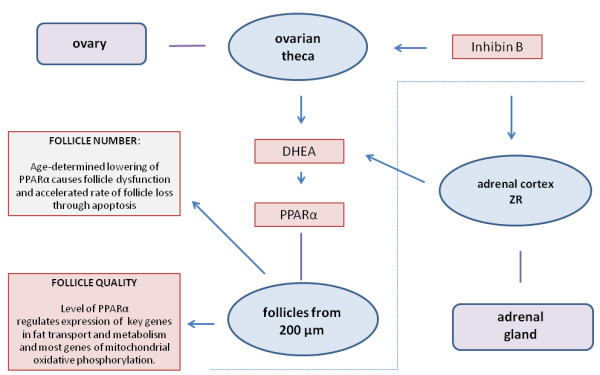
**The model (left and above dotted line) shows the response of the pre**-**antral theca to inhibin B, which results in production of DHEA and PPARα in follicles from about 200 μm.** It is proposed that reduced levels of PPARα primarily account for both the decline in follicle number and the loss of oocyte quality that ultimately leads to errors of cell division and arrested development. The defects that occur in cytoplasmic organelles, especially mitochondria, are largely responsible for decline in follicle quality. These can be attributed to key changes in fat metabolism and transport and mitochondrial function that are directly caused by dysfunction of PPARα secondary to low DHEA. These same changes also lead to increased production of ceramide, apoptosis and hence accelerated decline in the size of the follicle pool. The area to the right of the dotted line indicates that in humans and those primates where the adrenal ZR produces an alternative source of DHEA, that age related loss of ZR cells accelerates the rate of aging in the late thirties age group.

From the collective studies summarised in this review, it is concluded that mammalian female reproductive aging is caused by the gradual depletion of follicles which, through actions of lowered levels of inhibin B acting on the theca cells at the advanced secondary follicle stage, results in lowered levels of ovarian synthesis of DHEA. This in turn leads to reduction in the activity of many key metabolic systems. Both androgens and estrogens are affected but it is proposed here that lowered activation of PPARα is paramount because of its role in the activation of key enzymes affecting fatty acid synthesis and mitochondrial oxidative phosphorylation. Its effect on the down-regulation of the fatty acid enzyme delta-9 desaturase accounts for increased production of ceramide with aging and consequent increased rates of atresia. The model, which has been developed from the findings is depicted in Figure 2. It accounts for both the loss of follicles, and correlated decrease in quality that occur at a consistent exponential rate with aging in most mammals. Consequently, as well as increasing steroid levels, treatment with DHEA both reduces ovarian oxidative stress and rates of atresia.

In humans and some primates that undergo adrenarche, DHEA is also produced by the specialised area of the adrenal cortex known as the zona reticularus (ZR). The number of ZR cells declines markedly with age and the synthesis of adrenal DHEA is greatly affected by aging. Whether the lowered levels of inhibin B play a role in the age-related reduction of synthesis of adrenal DHEA or whether this is entirely independent and caused only by the reduction of ZR cells needs to be determined. As outlined in Figure 2, it is proposed that the decline in ZR function in humans with aging accounts for the accelerated decline in the quality of reproduction and increased rate of follicle loss that occurs in women after about age 37.

## Competing interest

The author declares that they have no competing interests.

## References

[B1] Carothers AD Castilla EE Dutra MG Hook EB Search for ethnic, geographic, and other factors in the epidemiology of Down syndrome in South America: analysis of data from the ECLAMC project, 1967–1997 Am J Med Genet 2001 20 2 149 156 11568922 10.1002/1096-8628(20011001)103:2<149::aid-ajmg1525>3.0.co;2-g

[B2] Hassold TJ Jacobs PA Trisomy in man Annu Rev Genet 1984 20 69 97 6241455 10.1146/annurev.ge.18.120184.000441

[B3] Nagaoka SI Hassold TJ Hunt PA Human aneuploidy: mechanisms and new insights into an age-old problem Nat Rev Genet 2012 20 7 493 504 22705668 10.1038/nrg3245 PMC3551553

[B4] Hultén MA Patel S Jonasson J Iwarsson E On the origin of the maternal age effect in trisomy 21 Down syndrome: the Oocyte Mosaicism Selection model Reproduction 2010 20 1 1 9 19755486 10.1530/REP-09-0088

[B5] Warburton D Biological aging and the etiology of aneuploidy Cytogenet Genome Res 2005 20 3–4 266 272 16192704 10.1159/000086899

[B6] Ford JH Wilkin HZ Thomas P McCarthy C A 13-year cytogenetic study of spontaneous abortion: clinical applications of testing Aust N Z J Obstet Gynaecol 1996 20 3 314 318 8883759 10.1111/j.1479-828x.1996.tb02719.x

[B7] Poland BJ Miller JR Harris M Livingston J Spontaneous abortion. A study of 1,961 women and their conceptuses Acta Obstet Gynecol Scand Suppl 1981 20 1 32 6952743

[B8] de Bruin JP Dorland M Spek ER Posthuma G van Haaften M Looman CW te Velde ER Age-related changes in the ultrastructure of the resting follicle pool in human ovaries Biol Reprod 2004 20 2 419 424 14561658 10.1095/biolreprod.103.015784

[B9] Tatone C Carbone MC Falone S Aimola P Giardinelli A Caserta D Marci R Pandolfi A Ragnelli AM Amicarelli F Age-dependent changes in the expression of superoxide dismutases and catalase are associated with ultrastructural modifications in human granulosa cells Mol Hum Reprod 2006 20 11 655 660 17005595 10.1093/molehr/gal080

[B10] Kushnir VA Ludaway T Russ RB Fields EJ Koczor C Lewis W Reproductive aging is associated with decreased mitochondrial abundance and altered structure in murine oocytes J Assist Reprod Genet 2012 20 7 637 642 22527902 10.1007/s10815-012-9771-5 PMC3401248

[B11] Sugimura S Matoba S Hashiyada Y Aikawa Y Ohtake M Matsuda H Kobayashi S Konishi K Imai K Oxidative phosphorylation-linked respiration in individual bovine oocytes J Reprod Dev 2012 20 6 636 641 22785440 10.1262/jrd.2012-082

[B12] Simsek-Duran F Li F Ford W Swanson RJ Jones HW Castora FJ Age-associated metabolic and morphologic changes in mitochondria of individual mouse and hamster oocytes PLoS One 2013 20 5 e64955 23741435 10.1371/journal.pone.0064955 PMC3669215

[B13] Van Blerkom J Davis PW Lee J ATP content of human oocytes and developmental potential and outcome after in-vitro fertilization and embryo transfer Hum Reprod 1995 20 2 415 424 7769073 10.1093/oxfordjournals.humrep.a135954

[B14] Feuerstein P Puard V Chevalier C Teusan R Cadoret V Guerif F Houlgatte R Royere D Genomic assessment of human cumulus cell marker genes as predictors of oocyte developmental competence: impact of various experimental factors PLoS One 2012 20 7 e40449 22848380 10.1371/journal.pone.0040449 PMC3407221

[B15] Feuerstein P Cadoret V Dalbies-Tran R Guerif F Bidault R Royere D Gene expression in human cumulus cells: one approach to oocyte competence Hum Reprod 2007 20 12 3069 3077 17951581 10.1093/humrep/dem336

[B16] McReynolds S Dzieciatkowska M McCallie BR Mitchell SD Stevens J Hansen K Schoolcraft WB Katz-Jaffe MG Impact of maternal aging on the molecular signature of human cumulus cells Fertil Steril 2012 20 6 1574 1580 e1575 22968048 10.1016/j.fertnstert.2012.08.012

[B17] Casson PR Lindsay MS Pisarska MD Carson SA Buster JE Dehydroepiandrosterone supplementation augments ovarian stimulation in poor responders: a case series Hum Reprod 2000 20 10 2129 2132 11006185 10.1093/humrep/15.10.2129

[B18] Gleicher N Weghofer A Barad DH The role of androgens in follicle maturation and ovulation induction: friend or foe of infertility treatment? Reprod Biol Endocrinol 2011 20 116 21849061 10.1186/1477-7827-9-116 PMC3170254

[B19] Fouany MR Sharara FI Is there a role for DHEA supplementation in women with diminished ovarian reserve? J Assist Reprod Genet 2013 20 9 1239 1244 doi: 10.1007/s10815-013-0018-x 6 23737215 10.1007/s10815-013-0018-x PMC3800538

[B20] Artini PG Simi G Ruggiero M Pinelli S Di Berardino OM Papini F Papini S Monteleone P Cela V DHEA supplementation improves follicular microenviroment in poor responder patients Gynecol Endocrinol 2012 20 9 669 673 22835219 10.3109/09513590.2012.705386

[B21] Hyman JH Margalioth EJ Rabinowitz R Tsafrir A Gal M Alerhand S Algur N Eldar-Geva T DHEA supplementation may improve IVF outcome in poor responders: a proposed mechanism Eur J Obstet Gynecol Reprod Biol 2013 20 1 49 53 23312476 10.1016/j.ejogrb.2012.12.017

[B22] Young JM McNeilly AS Theca: the forgotten cell of the ovarian follicle Reproduction 2010 20 4 489 504 20628033 10.1530/REP-10-0094

[B23] Ethun KF Wood CE Parker CR Kaplan JR Chen H Appt SE Effect of ovarian aging on androgen biosynthesis in a cynomolgus macaque model Climacteric 2012 20 1 82 92 21864136 10.3109/13697137.2011.571321 PMC3668306

[B24] Sasano H Okamoto M Mason JI Simpson ER Mendelson CR Sasano N Silverberg SG Immunolocalization of aromatase, 17 alpha-hydroxylase and side-chain-cleavage cytochromes P-450 in the human ovary J Reprod Fertil 1989 20 1 163 169 2644425 10.1530/jrf.0.0850163

[B25] Tamura T Kitawaki J Yamamoto T Osawa Y Kominami S Takemori S Okada H Immunohistochemical localization of 17 alpha-hydroxylase/C17-20 lyase and aromatase cytochrome P-450 in the human ovary during the menstrual cycle J Endocrinol 1992 20 3 589 595 1487710 10.1677/joe.0.1350589

[B26] Komar CM Peroxisome proliferator-activated receptors (PPARs) and ovarian function–implications for regulating steroidogenesis, differentiation, and tissue remodeling Reprod Biol Endocrinol 2005 20 41 16131403 10.1186/1477-7827-3-41 PMC1266036

[B27] Peters JM Zhou YC Ram PA Lee SS Gonzalez FJ Waxman DJ Peroxisome proliferator-activated receptor alpha required for gene induction by dehydroepiandrosterone-3 beta-sulfate Mol Pharmacol 1996 20 1 67 74 8700121

[B28] Feige JN Gelman L Michalik L Desvergne B Wahli W From molecular action to physiological outputs: peroxisome proliferator-activated receptors are nuclear receptors at the crossroads of key cellular functions Prog Lipid Res 2006 20 2 120 159 16476485 10.1016/j.plipres.2005.12.002

[B29] Goto T Lee JY Teraminami A Kim YI Hirai S Uemura T Inoue H Takahashi N Kawada T Activation of peroxisome proliferator-activated receptor-alpha stimulates both differentiation and fatty acid oxidation in adipocytes J Lipid Res 2011 20 5 873 884 21324916 10.1194/jlr.M011320 PMC3073464

[B30] Mandard S Müller M Kersten S Peroxisome proliferator-activated receptor alpha target genes Cell Mol Life Sci 2004 20 4 393 416 14999402 10.1007/s00018-003-3216-3 PMC11138883

[B31] Rakhshandehroo M Knoch B Müller M Kersten S Peroxisome proliferator-activated receptor alpha target genes PPAR Res 2010 doi: 10.1155/2010/612089 10.1155/2010/612089 PMC2948931 20936127

[B32] Patel MA Katyare SS Dehydroepiandrosterone (DHEA) treatment stimulates oxidative energy metabolism in the cerebral mitochondria from developing rats Int J Dev Neurosci 2006 20 5 327 334 16777366 10.1016/j.ijdevneu.2006.04.005

[B33] Jansen S Cashman K Thompson JG Pantaleon M Kaye PL Glucose deprivation, oxidative stress and peroxisome proliferator-activated receptor-alpha (PPARA) cause peroxisome proliferation in preimplantation mouse embryos Reproduction 2009 20 3 493 505 19531609 10.1530/REP-09-0038

[B34] Kujjo LL Acton BM Perkins GA Ellisman MH D’Estaing SG Casper RF Jurisicova A Perez GI Ceramide and its transport protein (CERT) contribute to deterioration of mitochondrial structure and function in aging oocytes Mech Ageing Dev 2013 20 1–2 43 52 23246342 10.1016/j.mad.2012.12.001

[B35] Perez GI Jurisicova A Matikainen T Moriyama T Kim MR Takai Y Pru JK Kolesnick RN Tilly JL A central role for ceramide in the age-related acceleration of apoptosis in the female germline FASEB J 2005 20 7 860 862 15728664 10.1096/fj.04-2903fje

[B36] Hu W Ross J Geng T Brice SE Cowart LA Differential regulation of dihydroceramide desaturase by palmitate versus monounsaturated fatty acids: implications for insulin resistance J Biol Chem 2011 20 19 16596 16605 21454530 10.1074/jbc.M110.186916 PMC3089502

[B37] Gougeon A Ovarian follicular growth in humans: ovarian ageing and population of growing follicles Maturitas 1998 20 2 137 142 9871908 10.1016/s0378-5122(98)00069-3

[B38] Penrose LS Mongolism Br Med Bull 1961 20 184 189 13734151 10.1093/oxfordjournals.bmb.a069906

[B39] Hook EB Rates of chromosome abnormalities at different maternal ages Obstet Gynecol 1981 20 3 282 285 6455611

[B40] Faddy MJ Gosden RG A model conforming the decline in follicle numbers to the age of menopause in women Hum Reprod 1996 20 7 1484 1486 8671489 10.1093/oxfordjournals.humrep.a019422

[B41] Faddy MJ Follicle dynamics during ovarian ageing Mol Cell Endocrinol 2000 20 1–2 43 48 10963872 10.1016/s0303-7207(99)00238-5

[B42] Hansen KR Knowlton NS Thyer AC Charleston JS Soules MR Klein NA A new model of reproductive aging: the decline in ovarian non-growing follicle number from birth to menopause Hum Reprod 2008 20 3 699 708 18192670 10.1093/humrep/dem408

[B43] Coxworth JE Hawkes K Ovarian follicle loss in humans and mice: lessons from statistical model comparison Hum Reprod 2010 20 7 1796 1805 20504871 10.1093/humrep/deq136

[B44] Faddy MJ Gosden RG Edwards RG Ovarian follicle dynamics in mice: a comparative study of three inbred strains and an F1 hybrid J Endocrinol 1983 20 1 23 33 6822780 10.1677/joe.0.0960023

[B45] Ingraham HA Hirokawa Y Roberts LM Mellon SH McGee E Nachtigal MW Visser JA Autocrine and paracrine Müllerian inhibiting substance hormone signaling in reproduction Recent Prog Horm Res 2000 20 53 67 discussion 67–58 11036933

[B46] Gruijters MJ Visser JA Durlinger AL Themmen AP Anti-Müllerian hormone and its role in ovarian function Mol Cell Endocrinol 2003 20 1–2 85 90 14656480 10.1016/j.mce.2003.09.024

[B47] Broekmans FJ Kwee J Hendriks DJ Mol BW Lambalk CB A systematic review of tests predicting ovarian reserve and IVF outcome Hum Reprod Update 2006 20 6 685 718 16891297 10.1093/humupd/dml034

[B48] Klein NA Houmard BS Hansen KR Woodruff TK Sluss PM Bremner WJ Soules MR Age-related analysis of inhibin A, inhibin B, and activin a relative to the intercycle monotropic follicle-stimulating hormone rise in normal ovulatory women J Clin Endocrinol Metab 2004 20 6 2977 2981 15181087 10.1210/jc.2003-031515

[B49] Welt CK McNicholl DJ Taylor AE Hall JE Female reproductive aging is marked by decreased secretion of dimeric inhibin J Clin Endocrinol Metab 1999 20 1 105 111 9920069 10.1210/jcem.84.1.5381

[B50] Hummer L Nielsen MD Christiansen C An easy and reliable radioimmunoassay of serum androstenedione: age-related normal values in 252 females aged 2 to 70 years Scand J Clin Lab Invest 1983 20 4 301 306 6635535

[B51] Wickenheisser JK Nelson-DeGrave VL McAllister JM Human ovarian theca cells in culture Trends Endocrinol Metab 2006 20 2 65 71 16460956 10.1016/j.tem.2006.01.003

[B52] Welt CK Schneyer AL Differential regulation of inhibin B and inhibin a by follicle-stimulating hormone and local growth factors in human granulosa cells from small antral follicles J Clin Endocrinol Metab 2001 20 1 330 336 11232020 10.1210/jcem.86.1.7107

[B53] Findlay JK An update on the roles of inhibin, activin, and follistatin as local regulators of folliculogenesis Biol Reprod 1993 20 1 15 23 8418903 10.1095/biolreprod48.1.15

[B54] Shelling AN Burton KA Chand AL van Ee CC France JT Farquhar CM Milsom SR Love DR Gersak K Aittomäki K Winship IM Inhibin: a candidate gene for premature ovarian failure Hum Reprod 2000 20 12 2644 2649 11098038 10.1093/humrep/15.12.2644

[B55] Woodruff TK Mather JP Inhibin, activin and the female reproductive axis Annu Rev Physiol 1995 20 219 244 7778866 10.1146/annurev.ph.57.030195.001251

[B56] Burger HG Hale GE Dennerstein L Robertson DM Cycle and hormone changes during perimenopause: the key role of ovarian function Menopause 2008 20 4 Pt 1 603 612 18574431 10.1097/gme.0b013e318174ea4d

[B57] Rainey WE Carr BR Sasano H Suzuki T Mason JI Dissecting human adrenal androgen production Trends Endocrinol Metab 2002 20 6 234 239 12128283 10.1016/s1043-2760(02)00609-4

[B58] Labrie F Bélanger A Cusan L Gomez JL Candas B Marked decline in serum concentrations of adrenal C19 sex steroid precursors and conjugated androgen metabolites during aging J Clin Endocrinol Metab 1997 20 8 2396 2402 9253307 10.1210/jcem.82.8.4160

[B59] Hornsby PJ Aging of the human adrenal cortex Sci Aging Knowledge Environ 2004 20 35 re6 15342924 10.1126/sageke.2004.35.re6

[B60] Vänttinen T Liu J Kuulasmaa T Kivinen P Voutilainen R Expression of activin/inhibin signaling components in the human adrenal gland and the effects of activins and inhibins on adrenocortical steroidogenesis and apoptosis J Endocrinol 2003 20 3 479 489 12967339 10.1677/joe.0.1780479

[B61] Arola J Liu J Heikkilä P Ilvesmäki V Salmenkivi K Voutilainen R Kahri AI Expression of inhibin alpha in adrenocortical tumours reflects the hormonal status of the neoplasm J Endocrinol 2000 20 2 223 229 10810286 10.1677/joe.0.1650223

[B62] Abraham GE Chakmakjian ZH Serum steroid levels during the menstrual cycle in a bilaterally adrenalectomized woman J Clin Endocrinol Metab 1973 20 4 581 587 4270264 10.1210/jcem-37-4-581

[B63] Abraham GE Ovarian and adrenal contribution to peripheral androgens during the menstrual cycle J Clin Endocrinol Metab 1974 20 2 340 346 4278727 10.1210/jcem-39-2-340

[B64] Fogle RH Stanczyk FZ Zhang X Paulson RJ Ovarian androgen production in postmenopausal women J Clin Endocrinol Metab 2007 20 8 3040 3043 17519304 10.1210/jc.2007-0581

[B65] Haning RV Hackett RJ Flood CA Loughlin JS Zhao QY Longcope C Plasma dehydroepiandrosterone sulfate serves as a prehormone for 48% of follicular fluid testosterone during treatment with menotropins J Clin Endocrinol Metab 1993 20 5 1301 1307 8496321 10.1210/jcem.76.5.8496321

